# Monteggia-like lesions in adults treated with radial head arthroplasty—mid-term follow-up of 27 cases

**DOI:** 10.1186/s13018-019-1540-z

**Published:** 2020-01-03

**Authors:** Matthias Jung, Corinna Groetzner-Schmidt, Felix Porschke, Paul A. Grützner, Thorsten Guehring, Marc Schnetzke

**Affiliations:** 1 Clinic for Trauma and Orthopaedic Surgery, BG Trauma Center Ludwigshafen at the University of Heidelberg, Ludwig-Guttmann-Strasse 13, 67071 Ludwigshafen on the Rhine, Germany; 2 Department for Shoulder and Elbow Surgery, Arcus Clinic Pforzheim, Rastatter Str. 17-19, 75179 Pforzheim, Germany

**Keywords:** Monteggia-like lesions, Radial head replacement, Radiographic evaluation, Operative therapy, Monteggia, Mason

## Abstract

**Background:**

The aim of the study was to analyze the functional and radiological outcome of Monteggia-like lesions in adults with unreconstructible fracture of the radial head and treatment with radial head arthroplasty.

**Methods:**

Twenty-seven patients (mean age 56 years; range 36 to 79 years) with a Monteggia-like lesion and treatment with radial head replacement were included in this retrospective study. Minimum follow-up was 2 years. Clinical assessment included the pain level with the visual analog scale in rest (VAS_R_) and under pressure (VAS_P_), range of motion, Mayo Elbow Performance Score (MEPS), and Disability of the Arm, Shoulder, and Hand score (DASH). A detailed radiological evaluation was performed. Complications and revisions were also analyzed.

**Results:**

After a mean follow-up period of 69 months (range, 24 to 170) the mean DASH score was 30 ± 24, the MEPS averaged 77 ± 20 points, the mean VAS_R_ was 2.1 ± 2.4, and VAS_P_ was 4.5 ± 3.5. Mean loss of extension was 24° ± 18 and flexion was 124° ± 20. Heterotopic ossifications were noted in 12 patients (44%). A total of 17 complications were noted in 11 patients (41%), leading to 15 revision surgeries in 9 patients (33%). Patients with a complicated postoperative course showed a worse clinical outcome compared with patients without complications measured by MEPS (68 ± 22 vs. 84 ± 16), DASH (49 ± 16 vs. 20 ± 22) and ulnohumeral motion (77° ± 31 vs. 117° ± 23).

**Conclusions:**

Monteggia-like lesions with unreconstructible radial head fracture and treatment with radial head replacement are prone to complications and revisions.

## Background

A Monteggia fracture consists by definition of a fracture of the ulna with ligamentous failure of the proximal radius resulting in dislocation of the radial head [1, 2]. The Monteggia fracture can be considered a rare injury, as it accounts for only 2 to 5% of all proximal forearm fractures [3]. The Monteggia-like lesion, a variant of the Monteggia fracture with a fracture of the radial head, is even rarer [4]. Monteggia-like lesions are severe injuries of the elbow with damage to stabilizing key structures of the elbow, such as the radial head and the coronoid process [5, 6]. Reports mostly focus on the treatment and the outcome of Monteggia-like lesions in children, which are distinct from those in adults with regard to the mechanism and patterns of injury [7]. Therefore, Monteggia-like lesions in children and adults should be considered separately. Even though there is a good understanding of the biomechanics of this type of fracture in adults, the rate of complications, revisions, and disappointing functional outcome results are high [5, 7–13]. If the Monteggia-like lesion is accompanied by unreconstructible radial head fractures, prosthetic replacement of the radial head is mostly recommended to prevent proximal migration of the radius [14, 15]. In previous studies, the patients treated with radial head replacement for Monteggia-like lesions were grouped with patients with other types of injuries, such as isolated radial head fractures or terrible triad injuries [16, 17]. The present study aimed to analyze the clinical and radiological results of Monteggia-like lesions in adults with unreconstructible radial head fractures and treatment with radial head replacement.

## Methods

Between January 2001 and May 2014, all consecutive patients with Monteggia-like lesion and treatment with radial head replacement were included if they met the following inclusion criteria: (1) age ≥ 18 years; (2) Monteggia-like lesion with unreconstructible radial head fracture, where anatomical reconstruction of the radial head fracture was not possible (3) treatment with radial head replacement (Evolve, Wright Medical Technology, Arlington, Tennessee); (4) minimum clinical and radiological examination of 2 years; (5) written informed consent. Patients with pre-existing elbow disorders and open fractures were excluded. In total, 27 of 37 patients (73%) could be enrolled. 10 patients (27%) were not able to participate in the follow-up examination at a minimum follow-up of 24 months, as they could not be reached for follow-up. Eleven patients were male (41%) and 16 were female (59%). The mean age of the study population was 56 years (36 to 79 years). Twenty-one patients had pre-existing conditions, and arterial hypertension was the most common (11 patients, 29%). In 11 patients, the dominant elbow was injured (41%).

According to the Bado classification [4], there were 4 patients with Bado Type I (15%), 22 patients with Bado Type II (81%), and 1 patient with a Bado Type III injury (4%). 22 patients with Bado Type II injuries were further classified according to Jupiter [18]. In 5 patients, there was a Type II A injury (23%), in 11 patients there was a Type II B injury (50%), 1 patient had Type II C injury (5%), and in 5 patients, there was an injury Type II D (23%). The coronoid fracture was classified according to Regan and Morrey [19], and 1 patient a Type II fracture (4%), and 9 patients a Type III fracture (33%). The radial head fractures were classified according to Mason [20] and all patients had a Mason Type III injury (100%).

### Operative care and rehabilitation

Surgery was performed after a mean of 3.5 days (range, 0 to 10 days). The operations were performed by three senior consultants from the Department of Shoulder and Elbow Surgery. In 16 patients (59%), primary surgery was not performed at the day of injury. In these cases, closed reduction was performed under general anesthesia or sedation, and successful reduction was confirmed under fluoroscopy. In 3 patients (11%), the elbow was primarily treated with closed reduction and external fixation with definitive treatment after 4, 6, and 10 days, respectively. The fracture of the proximal ulna was fixed with a proximally contoured 3.5-mm LCP (locking compression plate, Synthes GmbH, Umkirch, Germany) in 24 patients (89%) and tension wire in 3 patients (11%). The unreconstructible radial head fracture was treated with a radial head prosthesis (Evolve, Wright Medical Technology, Arlington, Tennessee) in all patients. In 7 patients (26%), the lateral collateral ligament was reattached using an anchor. In the case of Regan and Morrey Type II and III fractures, the coronoid process was stabilized using lag screws, introduced either through the ulnar plate or independently after indirect reduction of the fracture. The rehabilitation took place from the second postoperative day. Physiotherapists supervised the postoperative exercises. After 6 weeks full load, active and passive stretching and strengthening exercises were allowed too. According to the standardized postoperative pain scheme, patients were given ibuprofen 600 mg three times daily for prophylaxis of heterotopic ossification for three weeks.

### Clinical evaluation

The functional result was assessed by determining the range of motion in terms of elbow flexion, elbow extension, forearm supination, and forearm pronation with a goniometer. The functional outcome of the elbow was assessed using the Mayo Elbow Performance Score (MEPS) [21]. At the follow-up visit, patients completed questionnaires: visual analog scale (0–10) for pain at rest (VAS_R_) and pain at activity (VAS_A_), disabilities of the arm, shoulder, and hand (DASH) score [22], and satisfaction (1 = very satisfied, 2 = satisfied, 3 = slightly satisfied, 4 = somewhat dissatisfied, 5 = dissatisfied, 6 = extremely dissatisfied).

### Radiographic evaluation

**Fig. 1:**
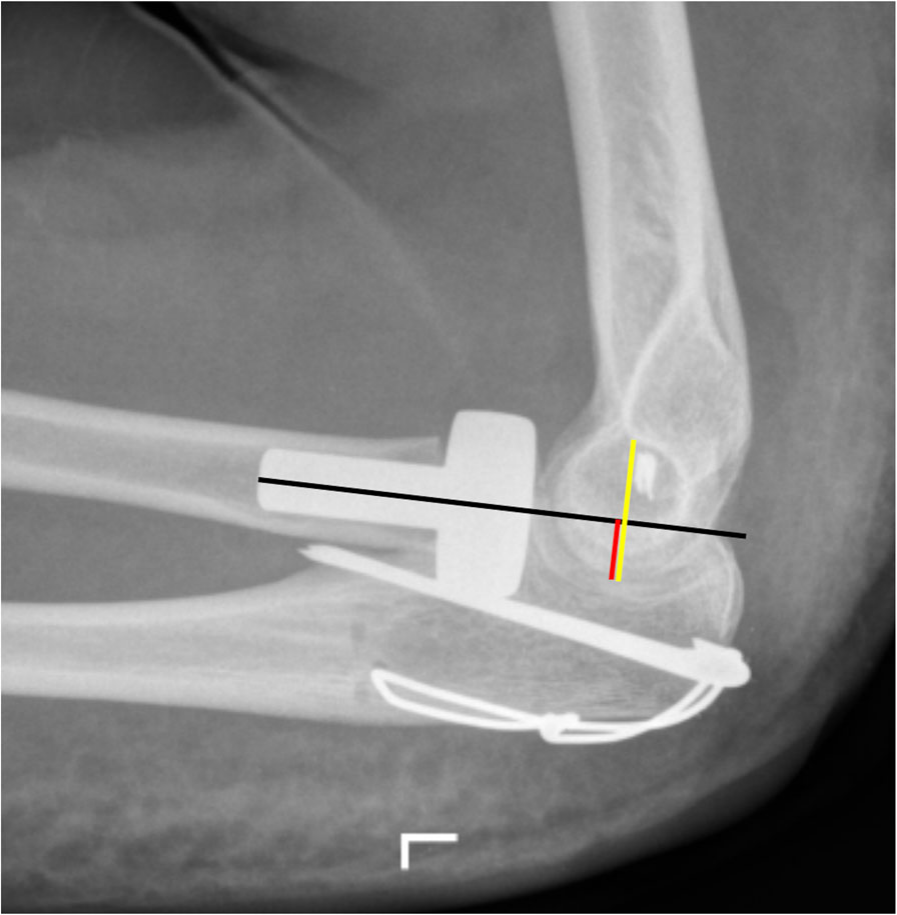
Radiocapitellar alignment was determined by the quotient of the red line and yellow line, which both run through the center of the capitellum and which are both perpendicular to the shaft axis of the stem (black line). For example, a quotient greater than 50% would indicate an anterior position of the radial head prosthesis

Standard anteroposterior and lateral radiographs of the elbow were performed preoperatively and at follow-up. Two surgeons (MS and MJ) evaluated the radiographs for (1) periprosthetic radiolucency, (2) radiocapitellar alignment of the radial head prothesis (Fig. 1), (3) ulnohumeral degeneration, (4) heterotopic ossification, and (5) osteopenia, and/or capitellar abrasion. Disagreements on the evaluation results were resolved by consensus. (1) The periprosthetic lucency around the shaft was determined according to the recommendation of Grewal et al. classified as non-mild, moderate or severe based on the number of zones and the amount of light observed [23]. (2) The position of the radial head prosthesis was assessed on the lateral radiograph image based on the intersection between the axis of the prosthetic shaft and the center of the capitellum. The calculation was based on the quotient of the diameter of the trochlea humeri and the axis of the prosthesis [24]. (3) The degree of ulnohumeral degeneration was classified with the system described by Broberg and Morrey as Grade 0 (normal joint), Grade 1 (mild degeneration), Grade 2 (moderate degeneration) or Grade 3 (severe degeneration) [8]. (4) Heterotopic ossifications were classified as present or absent. (5) Capitellar osteopenia and/or abrasion was classified as mild, moderate, or severe [16].

### Statistical analysis

Means and SDs were calculated for continuous variables. The Mann-Whitney *U* test was used to compare the two groups of patients. A 2-tailed *p* value of < 0.05 was considered to show a significant difference. In the analysis of contingency tables, the Pearson chi-square test (*n* ≥ 5) and the Fisher exact test (*n* < 5) were used. Also, the influence of five different factors (delayed definitive treatment > 24 h, Bado/Jupiter Type IIA/IID, Regan and Morrey Type II/III, heterotopic ossification, complication) on clinical outcome was assessed. Since the study was purely exploratory in design, and multiple tests without adjustment for multiplicity were performed, the reported p values can only be interpreted descriptively. SPSS software (version 23.0; IBM) was used for the analysis.

## Results

### Clinical results

After a mean follow-up of 69 months (range, 24 to 170) the MEPS averaged 77 ± 20 points and the mean DASH score was 30 ± 24 points. The mean flexion was 124° ± 20 and the mean loss of extension was 24° (±18°). The average ulnohumeral motion of the injured elbow was 100° ± 33. The supination could be performed at 67° (± 29°), the pronation at 64° (± 26°). The patients reported a mean VAS_R_ of 2.1 ± 2.4 and a mean VAS_A_ of 4.5 ± 3.4. Overall, there was a subjective satisfaction rate of 1.6 ± 1.1. Patients with lateral collateral ligament reconstruction (*n* = 7, 26%) showed comparable results as measured by MEPS (*p* = 0.282), DASH (*p* = 0.709), range of motion (*p* ≥ 0.174) and VAS (*p* ≥ 0.201).

### Radiographic results

**Fig. 2:**
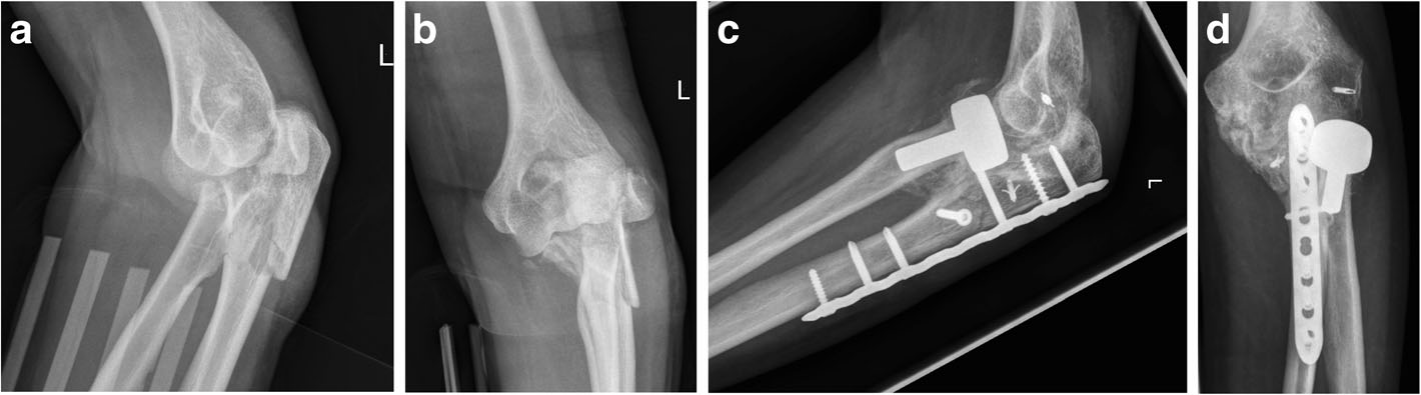
**a** Lateral and **b** anteroposterior radiographs of the left elbow of a 49-year-old woman shows a posterior Monteggia-like lesion (Bado Type II, Jupiter Type IIB), Mason Type III fracture of the radial head, and Regan and Morrey Type III coronoid fracture. **c**, **d** Six months after surgery, the patient was revised with open arthrolysis and removal of the heterotopic ossification due to a restriction of range of motion

In the radiographic assessment, 8 of 27 patients (30%) had no evidence of radiolucency around the stem. In 7 patients, the radiolucency was rated as mild (26%), in 5 patients as moderate (19%), and in 7 patients as severe (26%). Heterotopic ossifications around the elbow were observed in 12 patients (36%) (Fig. 2). The radiocapitellar alignment of the radial head prosthesis in lateral radiograph image was on average at 43% (range, 13 to 83). The detailed radiographic results are summarized in Table 1.

**Table 1:** Radiographic results of the study population

	*n* (%)
Periprosthetic radiolucency	
No	8 (30)
Mild	7 (26)
Moderate	5 (19)
Severe	7 (26)
Ulnohumeral degeneration	
No	11 (41)
Mild	12 (44)
Moderate	4 (15)
Severe	0
Capitellar abrasion	
No	8 (30)
Mild	14 (52)
Moderate	3 (11)
Severe	2 (7)

### Complications and revisions

Overall, 17 complications occurred in 11 patients (41%) leading to 15 revision surgeries in 9 patients (33%) (Tables 2 and 3). Three patients (11%) were revised three times. Most frequent complication was arthrofibrosis (*n* = 5; 19%) and overlengthening of the radial head prosthesis (*n* = 5; 19%). The prosthesis was exchanged in three patients (11%) due to oversizing and removed without replacement in another 3 patients (11%).

**Table 2:** Complications in the study population

	*n* (%)
Arthrofibrosis	5 (19)
Oversizing of the radial head prosthesis	5 (19)
Pseudarthrosis of the ulna	2 (7)
Secondary dislocation of the coroid process	2 (7)
Postoperative hematoma	1 (4)
Transient radial nerve palsy	1 (4)
Postoperative N. ulnaris syndrome	1 (4)

**Table 3:** Revision surgeries in the study population

	*n* (%)	Time to revision (*d*, range)
Open arthrolysis without removal of prosthesis	3 (11)	444 (82 to 1064)
Exchange of the prosthesis	3 (11)	24 (10 to 155)
Open arthrolysis with removal of prosthesis	3 (11)	1144 (427 to 1586)
Re-osteosynthesis of the ulna	2 (7)	80 (4 to 155)
Re-osteosynthesis of the coronoid process	2 (7)	83 (83)
Removal of hematoma	1 (4)	1
Neurolysis of the ulnar nerve	1 (4)	1334

### Risk factor analysis

A complicated postoperative course was associated with a deteriorated clinical outcome regarding MEPS (68 ± 22 vs. 84 ± 16; p = 0.061), DASH (49 ± 16 vs. 20 ± 22; p = 0.006), and ulnohumeral motion (77° ± 31 vs. 117° ± 23; p = 0.001). The presence of heterotopic ossifications was also associated with worse MEPS (68 ± 23 vs. 85 ± 14; p = 0.036). The type of injury (Bado/Jupiter Type IIA/IID; *p* ≥ 0.517) and delayed definitive treatment > 24 h after injury (p≥0.069) did not influence the clinical outcome (Table 4).

**Table 4:** Analysis of risk factors for deteriorated outcome

	Yes	No	*p* value
Delayed definitive treatment > 24 h	*n* = 16	*n* = 11	
MEPS	78 ± 20	76 ± 22	1.000
DASH	34 ± 28	24 ± 20	0.512
Ulnohumeral motion	91 ± 33	113 ± 28	0.069
Bado/Jupiter IIA/IID	*n* = 10	*n* = 17	
MEPS	77 ± 24	77 ± 18	0.776
DASH	28 ± 22	30 ± 26	0.971
Ulnohumeral motion	94 ± 39	104 ± 28	0.517
Heterotopic ossification	*n* = 12	*n* = 15	
MEPS	68 ± 23	85 ± 14	0.036
DASH	39 ± 27	21 ± 19	0.132
Ulnohumeral motion	92 ± 37	108 ± 27	0.322
Complication	*n* = 11	*n* = 16	
MEPS	68 ± 22	84 ± 16	0.061
DASH	49 ± 16	20 ± 22	0.006
Ulnohumeral motion	77 ± 31	117 ± 23	0.001

## Discussion

This study aimed to examine Monteggia-like lesion in adults who received radial head replacement for unreconstructible radial head fracture. After a mean follow-up of 69 months, the MEPS averaged 77 ± 20 points and the mean DASH score was 30 ± 24 points. The detailed analysis of the clinical results revealed that patients with a complicated postoperative course showed a trend towards worse results measured by MEPS (68 ± 22 vs. 84 ± 16) and significantly worse results regarding DASH (49 ± 16 vs. 20 ± 22) and ulnohumeral motion (77° ± 31 vs. 117° ± 23). In total, 11 of 27 patients (41%) developed complications, leading to 15 revision operations in 9 out of 27 patients (33%). Comparing these results with those in the literature is difficult because the outcome of Monteggia-like lesions in adults, and especially treatment with radial head arthroplasty, is scarcely reported in the literature.

Most recently, Jungbluth et al. reported on 62 adult patients with Monteggia-like lesion, and the authors found better results compared to the current study with regards to MEPS (91) and DASH (15) [15]. In the study of Jungbluth et al., the radial head fracture was classified as Mason Type III in 22 out of 62 patients, and these patients were treated with radial head replacement. Unfortunately, the outcome of those patients was not analyzed separately.

In 1998, Ring et al. investigated the outcome of 48 adult patients with Monteggia injuries after a mean follow-up of 6.5 years. Twenty-six were classified as Monteggia-like lesion with fracture of the radial head, and 12 patients had an unreconstructible radial head fracture, which was treated with resection in 10 patients and replacement with a silicone prosthesis in 2 patients. Ring et al. reported that 10 out of 12 patients, who had resection of the radial head without prosthetic replacement, had a satisfactory result. The outcome of the patients with radial head replacement was not reported. Although good clinical results were seen in most patients with Bado Type II fractures, Ring et al. reported that in 13 patients (50%), revision surgery was required within four months after primary surgery.

Konrad et al. evaluated Bado Type II fractures at a mean follow-up of eight years with an associated fracture of the radial head, of the coronoid process, or both in 11 patients [9]. The authors found that fractures of the coronoid process or radial head were risk factors for poor Broberg-Morrey scores.

Givon et al. investigated the outcome of 41 patients with Monteggia-like lesions (14 children and 27 adults) with an average follow-up of 4.8 years [25]. In 24 of 27 adult patients, the radial head fracture was treated with open reduction and internal fixation; 3 patients were treated non-operatively due to severely comminution. In agreement with Konrad et al., the authors were able to show that an additional fracture of the radius head was associated with a poor outcome. In adults, the complication rate was 24%, which is comparable to the finding in the current study.

In agreement with the literature, a high number of patients with heterotrophic ossifications (44%) were observed in the current study. In a similar study, colleagues Antonio et al. reported a rate of 37% for proximal forearm fractures treated surgically [26]. Distal humeral fracture, terrible triad injury, Monteggia-like lesion, open injury, instability, severe breast trauma, or delayed final surgical treatment have been identified as risk factors for the development of heterotopic ossifications. Egol et al. carried out a retrospective evaluation of the clinical outcomes of 20 patients with a fracture of the proximal ulna, radial head or neck, and dislocation of the radial head [5]. At a mean follow-up of 2.3 years, the mean Broberg-Morrey score was 79 and the mean DASH score was 64. This accounted for only 11 (55%) of the patients with good or excellent results. Heterotopic ossification was seen in 7 patients (35%) and arthritic changes in 14 (70%).

Schmalzl et al. assessed the outcome of 14 patients with Monteggia-like lesion with a mean follow-up of 22 months [27]. The authors reported a satisfactory MEPS (82) and DASH score (24). Three patients required surgical revision (23%). In the case of comminuted radial head fractures, the authors recommended replacement instead of resection due to a subsequent loss of stability. Unfortunately, the authors did not mention how many patients were treated with radial head replacement in this series.

In the current study, Monteggia-like lesion with an unreconstructible radial head fracture and prosthetic replacement of the radial head were associated with very high revision (33%) and complication rates (41%). Most of these were related to the radial head replacement: in 6 out of 15 revisions (40%), the radial head prosthesis was exchanged or removed. One major challenge of radial head replacement in Monteggia-like lesions might be the adjustment of the correct length of the prosthesis. The lateral ulnar joint line serves as the reference point for correct sizing of the radial head prosthesis [28]. By definition, the ulna is fractured in Monteggia-like lesions. Therefore, correct restoration of the length of the radial head relies on an anatomical reduction of the ulna. In the case of a fractured coronoid process, length and size planning of the radial head prosthesis is even more difficult [16, 29]. The literature shows that radial head excision is linked to inferior clinical results compared to open reduction and internal fixation or radial head replacement, as radiocapitellar contact is important for elbow and forearm stability [30, 31]. In 2017, Matar et al. reported the outcome of 22 patients treated for Monteggia-like lesion with a mean follow-up of 4.1 years [30]. In 9 patients, radial head replacement was performed and those patients had a mean MEPS of 78.3 ± 25.4, which is comparable to the results in the current study. In another 5 patients, patients were treated with excision of radial head fragment, which led to fair or poor outcome in 3 out of 5 patients. In 1997, Singh et al. reported the outcome of 6 patients with Monteggia-like lesions who received radial head resection, and 3 patients had a fair or poor result according to the MEPS [31]. Therefore, radial head excision is contraindicated in acute situations and should only be considered as a salvage procedure or for elderly patients with low functional demands in order to minimize operating time [32–34]. Successful treatment of these kinds of severe injuries depends on understanding all aspects of this injury, including the coronoid process, the radial head, and ligamentous injury. Therefore, a standardized approach with preoperative computerized tomography is necessary for preoperative planning. With anatomical reduction of the ulna and the coronoid process as well as restoration of the correct length of the radial head, good results can be achieved.

### Limitations

The present study is limited by its retrospective design and the small number of patients. Although only patients with Monteggia-like lesion and treatment with radial head prosthesis were included, the study population is very heterogeneous. In addition, the follow-up time (2 to 14 years) and the age of the patients (36 to 79 years) are very different and can be confounding factors that influence the results. Ten out of 37 patients (27%) were lost to follow-up and could not be included in the study. There was no control group and no performance analysis were performed. Also, the clinical and radiographic follow-up examinations were performed by the principal investigator and senior author, who were not blinded to patients’ history. This might have created detection bias.

## Conclusion

Monteggia-like lesions in adults with comminuted radial head fractures are severe injuries associated with high rates of complications and revisions. In the case of unreconstructible radial head fracture, the possibility of implanting a radial head prosthesis must be available. The operative surgeon should be aware that correct sizing of the radial head prosthesis in the context of Monteggia-like lesions is challenging due to fracture of the ulnar and/or the coronoid process.

## Data Availability

All data and materials regarding the study are available from the corresponding author.

## Abbreviations

DASH: Disability of the Arm, Shoulder, and Hand score
LCP: Locking compression plate
MEPS: Mayo Elbow Performance Score
VAS_P_: Visual analog scale under pressure
VAS_R_: Visual analog scale in rest
